# Open sharing of genomic data: Who does it and why?

**DOI:** 10.1371/journal.pone.0177158

**Published:** 2017-05-09

**Authors:** Tobias Haeusermann, Bastian Greshake, Alessandro Blasimme, Darja Irdam, Martin Richards, Effy Vayena

**Affiliations:** 1 Health Ethics and Policy Lab, Epidemiology, Biostatistics & Prevention Institute (EBPI), University of Zurich, Zurich, Switzerland; 2 Department of Sociology, University of Cambridge, Cambridge, United Kingdom; 3 Department for Applied Bioinformatics, Institute for Cell Biology and Neuroscience, Goethe University, Frankfurt am Main, Germany; 4 Centre for Family Research, Department of Psychology. University of Cambridge, Cambridge, United Kingdom; Columbia University Medical Center, UNITED STATES

## Abstract

We explored the characteristics and motivations of people who, having obtained their genetic or genomic data from Direct-To-Consumer genetic testing (DTC-GT) companies, voluntarily decide to share them on the publicly accessible web platform openSNP. The study is the first attempt to describe open data sharing activities undertaken by individuals without institutional oversight. In the paper we provide a detailed overview of the distribution of the demographic characteristics and motivations of people engaged in genetic or genomic open data sharing. The geographical distribution of the respondents showed the USA as dominant. There was no significant gender divide, the age distribution was broad, educational background varied and respondents with and without children were equally represented. Health, even though prominent, was not the respondents’ primary or only motivation to be tested. As to their motivations to openly share their data, 86.05% indicated wanting to learn about themselves as relevant, followed by contributing to the advancement of medical research (80.30%), improving the predictability of genetic testing (76.02%) and considering it fun to explore genotype and phenotype data (75.51%). Whereas most respondents were well aware of the privacy risks of their involvement in open genetic data sharing and considered the possibility of direct, personal repercussions troubling, they estimated the risk of this happening to be negligible. Our findings highlight the diversity of DTC-GT consumers who decide to openly share their data. Instead of focusing exclusively on health-related aspects of genetic testing and data sharing, our study emphasizes the importance of taking into account benefits and risks that stretch beyond the health spectrum. Our results thus lend further support to the call for a broader and multi-faceted conceptualization of genomic utility.

## Introduction

Genomic research promises major advances in our understanding of health and disease. In parallel, sharing genomic data offers encouraging prospects to accelerate research by generating information-rich genome datasets. Such benefits, however, will only reach the general population if researchers and clinicians can access, make comparisons and seek patterns across the genomes of a large number of individuals. Indeed, most studies require the aggregate of genomic data from large cohorts of individuals, both healthy and diseased, before any health-related utility can be established with reasonable confidence. However, numerous technical, legal, and ethical bottlenecks hamper the exploitation of such data [1]. Moreover, due to the diversity and fragmentation in health systems and medical databases, there is a lack of harmonization of data formats, processing, analysis and data transfer, which often leads to incompatibilities and lost opportunities for scientific advancement [2]. This difficulty in sharing genetic data for research purposes is aggravated by the fact that despite much progress, genomic and clinical data are still generally collected and studied in silos: by disease, by institution and by states [3] Although international guidelines have facilitated sharing and generally take a proportional risk approach, many countries have put in place strict provisions guiding international sharing, and a few even prohibit it entirely [4]. Furthermore, while experts support rigorous oversight and assessments of inter-lab reproducibility, they also caution that regulation should not pose an excessive administrative burden on academic and smaller diagnostic laboratories that are already saddled with burgeoning research expenses [5].

Despite such limitations, over the last few years, increasing amounts of genomic data have been generated and became available for research purposes through a number of different platforms. One of the first projects to address the need for large scale genomic data sharing was the Personal Genome Project (PGP), which aimed at enrolling 100,000 participants [6]. Launched in 2005 under the umbrella of the Harvard Medical School, its network has since expanded and now includes projects in Canada, the UK and Austria [7].The participants’ genomes are analysed by PGP and subsequent results, together with phenotypic data, are made public. Another source of genetic data stems from Direct-To-Consumer genetic testing (DTC-GT)–offered by companies like 23andMe, Family Tree DNA or Ancestry.com [8,9]. Thanks to the rapid growth of DTC-GT, data have become available for an increasingly broad audience over the last decade. One project making use of this data is dna.land, which began in 2015 at the New York Genome Center and to which DTC-GT costumers can upload their own data [10]. As a result, the dna.land team can use the data for research purposes whereas DTC-GT customers receive a genealogical analysis in return. The data and results, however, are not publicly accessible.

An alternative to the above approaches, likewise making use of the rising numbers of DTC-GTs, is openSNP [11]. Initiated in 2011, the platform allows individuals to contribute diverse sets of DTC-GT results, along with phenotypic annotations about themselves. Specifically, users can share their DTC-GT results from micro-array based genotyping, which makes up the vast majority of all data sets. Furthermore, users can upload VCF (variant call format) files, which may include exomes and full genomes. Genomic and phenotypic data are subsequently openly available to anyone, without any limits or restrictions on the use of the data. In that sense, the project combines both the open participation approach of dna.land and the open data functionality of the PGP. But unlike the PGP and dna.land, openSNP is not affiliated to an academic institution. Instead, it serves as a non-profit project that is run by a small group of volunteers and is funded through individual donations, collected via an ongoing crowdfunding campaign. Moreover, the licensing terms of openSNP do not restrict the reusing of genetic data, as the users share their data by accepting their release in the public domain through the Creative Commons Zero framework. On the openSNP website, the agreement thus states, “You agree that all data you upload to openSNP will be freely available online (well, except your mail-address and password) under a Creative Commons Zero license. The data can be viewed and downloaded through this webpage, RSS-feeds, and, in the future, perhaps via an API or FTP. Although you can delete your data from openSNP, this does not guarantee that someone else has not already created a backup of the data (who may re-publish the data somewhere else)” [12]. Despite this radically open approach, the project has attracted more than 5,000 registered users to date (2017-02-06), relying purely on social media and word of mouth for recruiting. Since September 2011, users have uploaded over 3,000 data sets [13] and interest in using them for scientific studies and commentary has continued to grow [14–17].

Given the concerns about genomic privacy, and specifically DTC-GT consumers’ privacy risks, openSNP presents a case of special interest. What is original about the openSNP approach is that it attracts users willing to openly share their genomic data while accepting that no protection of their privacy is on offer. Some users upload their data using pseudonyms; others use their real names, and some even link their social media profiles to their openSNP profiles. Research on DTC-GT consumers has so far focused on exploring motivations for genomic testing, the perception of risk by DTC-GT users and the impact of testing on consumers’ lives and behavior. And whereas earlier studies have also investigated the motivations and attitudes of individuals toward genetic and genomic data sharing [18–25], this is the first study of its kind to describe individuals who have decided to share their data publicly and without any institutional oversight. Our study describes openSNP users’ characteristics, their motivations for sharing as well as their views about privacy and explores what drives their choice to share their genomic data in the public domain.

## Materials and methods

In order to understand the openSNP users’ background, main interests and impetus to openly share their genomic data and their attitudes on issues of privacy, an anonymous online survey was emailed to 3884 openSNP users, yielding a 14.16% overall response rate (n = 550; 323 male, 221 female, 1 other, 5 n/a). This response rate is consistent with other recent online-based surveys [26]. The survey included both closed and open-ended questions on a) the respondents’ demographic information; b) their openness to and willingness to share genetic data; and c) the privacy-related concerns they might have regarding their data sharing or potential data sharing in the future. The respondents were offered a non-response (skip) option for all questions and analyses were conducted using descriptive and inferential statistics for the closed answers.

We further examined the associations between the respondents’ motivations to participate in genetic and genomic data sharing research and some of their basic demographic characteristics by using linear and logistic regression. Our model is based on a linear regression using a variable on how strongly the respondents feel about contributing to advancement of medical research as the dependent variable, controlling for such covariates as gender (male, female or other), having children and the respondents’ highest level of education (ranging across four categories from high school to doctorate levels). We used significance at the level of p < 0.05 in the analysis, which was conducted employing Stata/SE 12.0.

The Zurich Cantonal Ethics Commission waived review of this project as it involved research with anonymous data.

## Results

**Fig 1:**
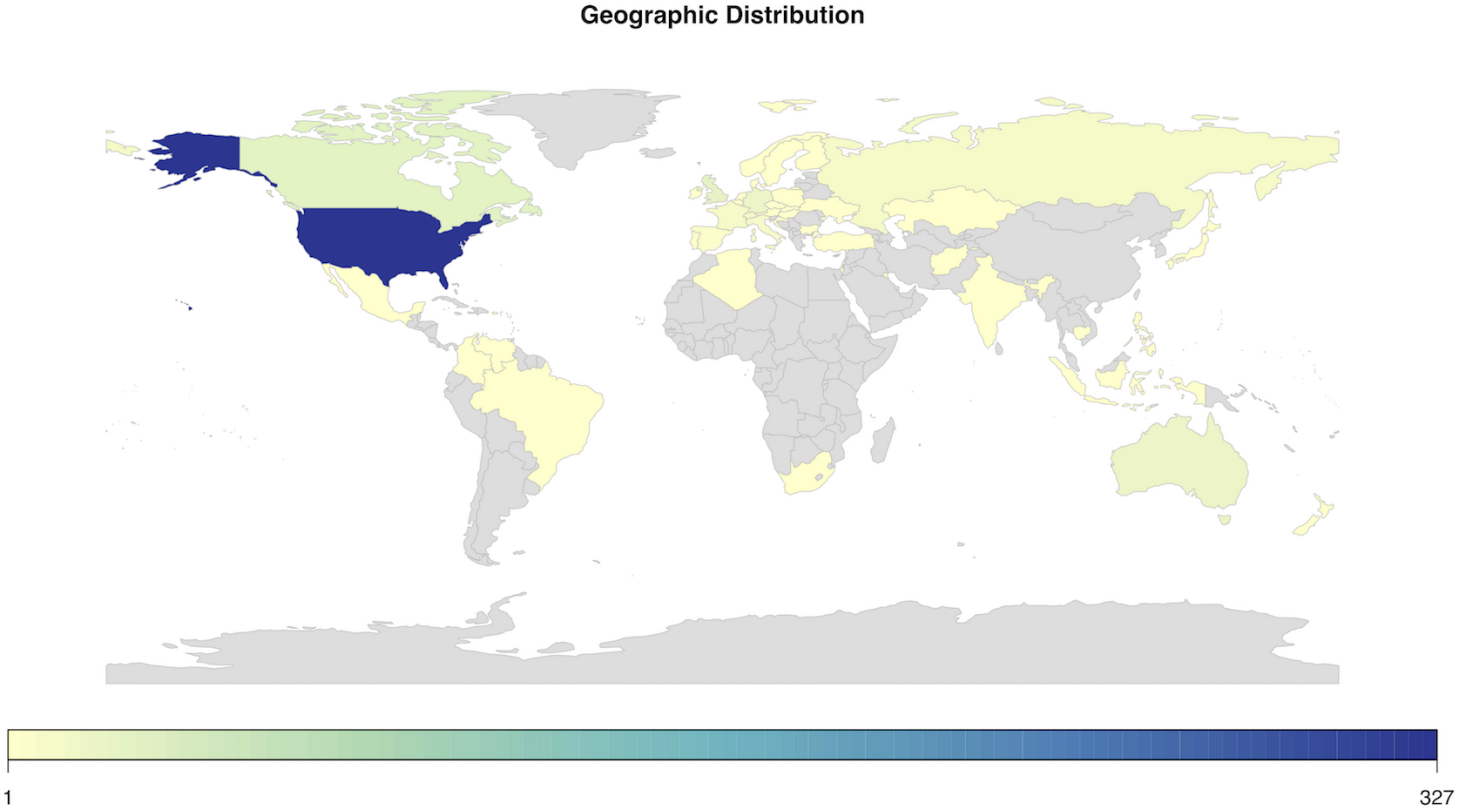
World map of survey population.

**Fig 2:**
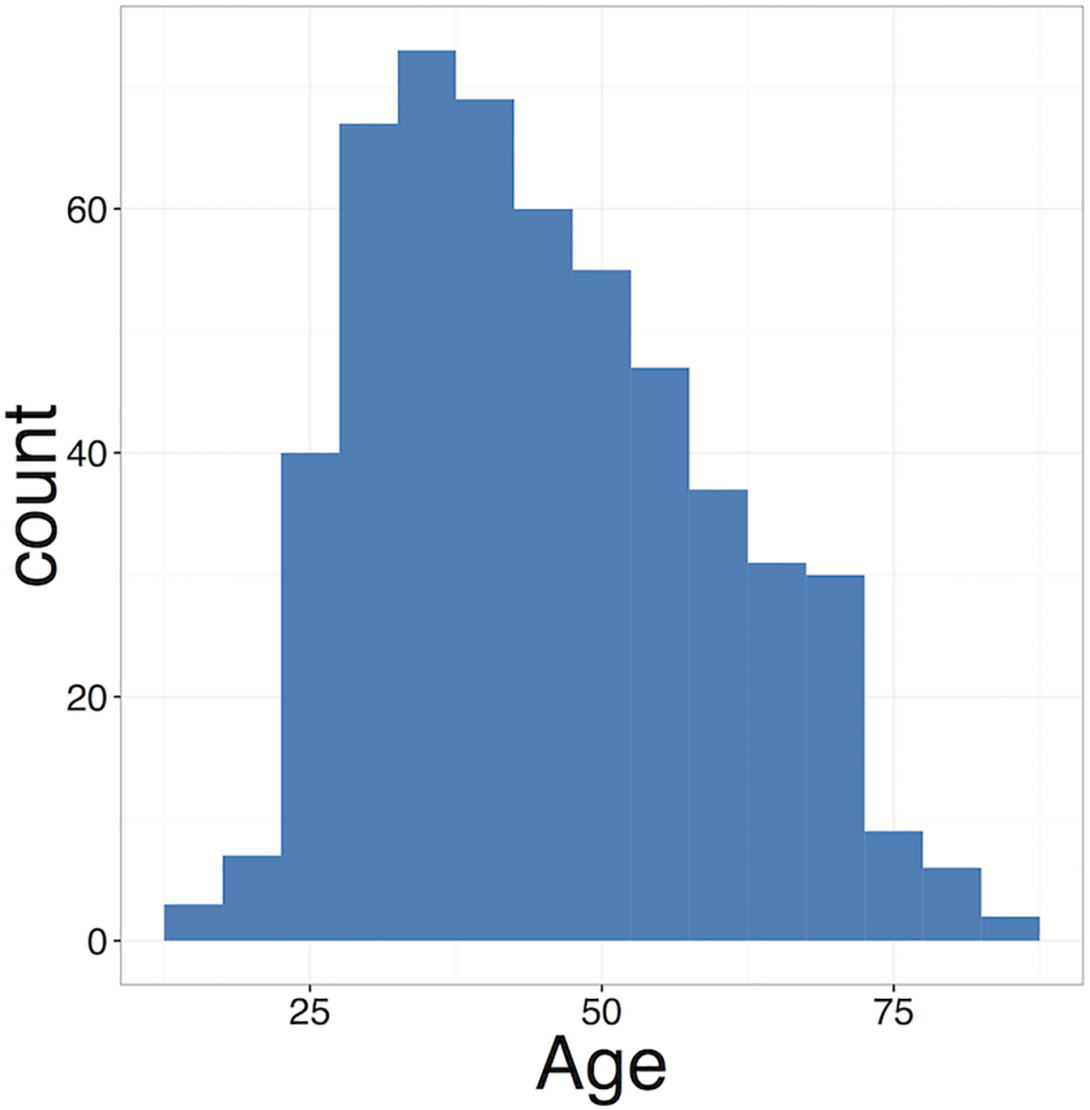
Age histogram of survey population.

The majority of respondents (60.33%) came from the USA, followed by Canada (5.17%) and the UK (4.61%). A further 7 countries each contributed between 4 and 1% of our respondents, namely Australia (3.32%), France (2.21%), Switzerland (2.21%), Russia (2.03%) and Italy (1.48%) (Fig 1). The remainder were scattered across some 41 countries, from all continents except Antarctica. The age distribution was broad, with the youngest respondents being born in 1995 and the oldest respondent in 1938 (Fig 2). 59.27% of respondents were men and 49.08% of all the respondents had children (see Table 1).

**Table 1:** Sample characteristics (n = 550).

**Gender (n)**	
**Male**	323
**Female**	221
**Other**	1
**Highest level of completed education (n)**	
**Pre-high school**	10
**High school**	113
**Bachelor’s degree**	210
**Postgraduate degree**	213
**Having children? (n)**	
Yes	268
No	278

Most respondents (74.30%) were not engaged with biomedical research; 15.57% were or had been research participants while 14.63% were involved in biomedical research in a professional capacity. The educational composition and engagement in biomedical research by gender is illustrated in Table 2.

**Table 2:** Participants’ education and engagement in biomedical research by gender.

**Education**	**Women (%)**	**Men (%)**	**Total (N)**
**Pre-high school**	0.45	2.80	10
**High school**	24.43	18.07	113
**Bachelor's degree**	45.70	33.64	210
**Postgraduate degree**	29,41	45,48	213
**Engaged in biomedical research…**	**Women (%)**	**Men (%)**	**Total (N)**
**…as a professional**	10.36	16.62	78
**…as a participant**	15.32	14.80	83
**Not engaged**	74.32	68.58	396

Many of the respondents had used more than one genome profiling and ancestry companies. 23andMe was the most popular with 412 users, followed by Ancestry.com and Family Tree DNA both with around 80 users. Numerous other platforms, including National Geographic and the Personal Genome Project, had been chosen, albeit to a considerably lesser degree. Participants were asked to rank three options for their interest in being tested: health, ancestry or as a contribution to research. As a result, 46.41% gave ancestry as their first option, followed by health (41.56%) whereas contribution to research trailed third (14.35%). Other reasons included genealogy, locating relatives and curiosity. When asked if DNA testing had helped them improve their health or life style, around a third checked not at all (33.20%) or to a small extent (38.49%) while 19.14% recorded a moderate and 9.16% a large improvement.

Concerning motivations to share genetic and genomic data on openSNP, our preliminary observational results show that 86.05% indicated wanting to learn about themselves as relevant or very relevant, followed by contributing to the advancement of medical research (80.30%), wanting to help improve the predictability of genetic testing and considering it fun to explore genotype and phenotype data (76.02% and 75.51%, respectively). The distribution of reasons to participate in genetic data sharing by gender is illustrated in Table 3.

**Table 3:** Motivation to participate in genetic data sharing by gender.

.		Not at all relevant	Somewhat relevant		Relevant / very relevant	Total
**Women (%)**	**I wanted to learn more about myself**	**1.41**		**9.15**	**89.44**	**100.00**
**Men (%)**		**3.54**		**12.63**	**83.83**	**100.00**
**Total (N)**		**9**		**38**	**293**	**340**
**Women (%)**	**Contribute to the advancement of medical research**	**5.63**		**12.68**	**81.69**	**100.00**
**Men (%)**		**6.19**		**14.43**	**79.38**	**100.00**
**Total (N)**		**20**		**46**	**270**	**336**
**Women (%)**	**Help improve the predictability of genetic testing**	**7.75**		**14.79**	**77.46**	**100.00**
**Men (%)**		**8.67**		**16.33**	**75**	**100.00**
**Total (N)**		**28**		**53**	**257**	**338**
**Women (%)**	**Consider it fun to explore genotype and phenotype data**	**6.34**		**19.72**	**73.94**	**100.00**
**Men (%)**		**8.63**		**14.72**	**76.65**	**100.00**
**Total (N)**		**26**		**57**	**256**	**339**

Most respondents had uploaded their genotypic raw data to openSNP fairly recently, in many cases in the previous few months. Of the 550 study subjects, 178 did not specify how often they had visited the website, whereas 101 (27.15%) reported they had only done so for uploading data and 181 (48.66%) a couple of times since then. Meanwhile, 78 (20.97%) stated that they visit occasionally, although only 12 respondents (3.23%) reported to visit the website regularly.

What is more, the respondents’ interest in uploading other data beyond their raw genotype data was limited. Only 35.83% of respondents had added phenotypes, but a further 49.72% thought they would do so in the future. Nonetheless, some users had created their own phenotypes by adding new trait questions to the openSNP database. Of the 5082 currently registered users, 1041 (20.48%) shared their eye color, 675 (13.28%) their hair color, 257 (5.06%) their political ideology, 40 (0.79%) their colon cancer status and 23 (0.45%) their status for hereditary breast and ovarian cancer [27].

In our basic exploratory model we also tested whether or not respondents identified their willingness to contribute to the advancement of medical research as their main priority for participating in genetic and genomic data sharing. Our model’s results are presented in Table 4, where we demonstrate that a) having children and b) the level of education have a significant effect on the relevance of the participants’ wish to contribute to medical research as a motivation for sharing their genetic data. Our results show that both having children and a master’s degree reduce the relevance of wanting to contribute to medical research in our participants.

**Table 4:** Having children and the level of education effect on the relevance of wanting to contribute to advancement of medical research among open SNP participants.

	Coef.	Robust SE	T	p value	95% Conf. Interval	
**Gender**	-0.13	0.10	-1.35	0.177	-0.33	0.06
**Having children**	-0.23	0.10	-2.30	0.022*	-0.43	-0.03
**Education**						
**High school**	-0.15	0.24	-0.63	0.531	-0.62	0.32
**Undergraduate**	-0.28	0.23	-1.22	0.222	-0.73	0.17
**Graduate**	-0.47	0.24	-1.97	0.050*	-0.94	0.00
**Doctorate**	-0.10	0.25	-0.38	0.706	-0.59	0.40

* N = 336, R-squared = 0.042

Since our explorative results indicate that education and having children render the contribution to the advancement of medical research less relevant for our participants’ motivation to participate in genetic and genomic data sharing, perhaps, future research attempts should be aimed at investigating how individuals’ motivation changes with increasing education or when having children.

With regard to privacy and confidentiality concerns, 73.17% of the respondents stated that they had not read or had only read quickly over the terms and conditions for sharing their data while registering on openSNP. Table 5 shows how accurately the participants read the terms and conditions of the openSNP platform by gender and education, respectively.

**Table 5:** How accurately the respondents have read the terms and conditions of the openSNP platform by gender and education (in %).

		Not at all	I only read them over Quickly	Carefully	Total
**By gender**	**Women (%)**	28.57	55.36	16.07	100
	**Men (%)**	51.43	32.86	15.71	100
	**Total (N)**	52	54	20	126
**By education**	**Pre-high school (%)**	0	0	100	100
	**High school (%)**	45	50	5	100
	**Bachelor's (%)**	38.78	42.86	18.37	100
	**Master's (%)**	35.71	50.00	14.29	100
	**PhD (%)**	50.00	32.14	17.86	100
	**Total (N)**	52	54	20	126

A large majority of participants thought it was rather likely (34.39%), or very likely (52.02%), that their genetic data could never be fully deleted. In addition, 65.99% of the respondents deemed it likely that their data will be used for purposes other than research. Nearly half of the respondents also thought it rather likely (34.69%) or very likely (13.12%) that their data would be employed for research of which the respondents themselves would not approve. Moreover, more than half of the respondents stated they would be not at all (26.45%) or rather little concerned (37.79%) with that prospect. However, the majority would be rather concerned (19.88%) or very concerned (38.60%), were an employer to use their genotype data to their disadvantage. Asking the same question with regard to insurance companies, 45.88% would be very concerned or at least rather concerned (23.24%), although the vast majority considered this to be a rather unlikely (38.89%) or very unlikely (48.54%) scenario in case of the employer, with similar results for the insurance company (rather unlikely 37.76% or very unlikely 27.14%). Table 6 presents an overview of the respondents’ views on privacy and genomic data protection.

**Table 6:** Respondents’ beliefs on the likelihood of data being fully deleted or misused by third parties and how concerned they would be about it.

	** **	**Very unlikely**	**Rather unlikely**	**Rather likely**	**Very likely**	**Total**
**Can your data never be fully deleted?**	**Percent**	2.31%	11.27%	34.39%	52.02%	100%
	**Absolute**	8	39	119	180	346
**Will your data be used to do research that you would not approve of?**	**Percent**	14.87%	37.32%	34.69%	13.12%	100%
	**Absolute**	51	128	119	45	343
**Will your data be used to your disadvantage by an employer?**	**Percent**	48.54%	38.89%	9.06%	3.51%	100%
	**Absolute**	166	133	31	12	342
**Will your data be used to your disadvantage by an insurance company?**	**Percent**	27.14%	37.76%	23.60%	11.50%	100%
	**Absolute**	92	128	80	39	339
**How concerned would you be about your data being used in different ways?**						
	**Not at all concerned**	**Rather not concerned**	**Rather concerned**	**Very concerned**	**Total**
**If your data would be used to do research you would not approve of?**	**Percent**	26.45%	37.79%	22.38%	13.37%	100%
	**Absolute**	91	130	77	46	344
**If your data would be used to your disadvantage by an employer?**	**Percent**	23.68%	17.84%	19.88%	38.60%	100%
	**Absolute**	81	61	68	132	342
**If your data would be used to your disadvantage by an insurance company?**	**Percent**	15.00%	15.88%	23.24%	45.88%	100%
	**Absolute**	51	54	79	156	340

## Discussion

The constantly decreasing cost of large-scale genotyping and genome sequencing has sustained the burgeoning growth of the personal genomic industry. This phenomenon has attracted a lot of research attention. While most empirical research on personal genomics has focused on the attitudes of early DTC-GT users, however, the present study aimed at shedding light on the characteristics and motivations of people who, having obtained their genetic or genomic data from a DTC-GT company, voluntarily decide to share them on a fully accessible platform like openSNP. Our findings highlight the diversity and range of DTC-GT consumers. The geographical distribution of the respondents showed the USA and other mainly English speaking countries as predominant. The age distribution was broad and there was no marked gender divide. Educational background was varied, with the median distribution towards a slightly more highly educated population. These characteristics do not corroborate other research, which suggests that, as a rule, individuals purchasing DTC genetic and genomic test are highly educated, middle aged users [28,29].

### Data sharing and genomic utility

The emergence of direct-to-consumer genetic and genomic tests seems to reflect the interest in genetic testing amongst a diverse array of users. Indeed, DTC-GT companies have rapidly generated growing consumer interest by advertising health-related and ancestry tests. Both in the case of tests providing health-related information and genetic ancestry testing, however, the scientific validity of the information released to consumers has been a major issue of debate. In the field of health-related DTC-GT, such controversies eventually contributed to the Food and Drug Administration (FDA) requesting 23andMe to discontinue their health-related Personal Genome Service in 2013, as the company had failed to provide answers about the analytical and clinical validity of their services [30]. Two years later, 23andMe gained approval from the FDA to market a carrier test for Bloom syndrome only [31] and the company subsequently went back on the market with one-quarter of their initial tests.

In light of these events, the risks and benefits of DTC health testing must be carefully weighed, as today they remain far from clear. By and large, the majority of arguments for and against direct-to-consumer genomic tests revolve around two issues: first, whether the tests bring any personal or clinical utility and, second, whether the commercial availability of such tests constitutes a threat to consumers or, rather, an opportunity for the cultivation of individual autonomy [32]. With regard to personal or clinical utility, preliminary findings suggest that the health benefits envisioned by DTC-GT companies (e.g. significant improvements in positive health behaviors) have not materialized to date [33,34]. Studies investigating the test data of the services further confirm that the tests have little predictive power and do not measure genetic risk appropriately [35]. Moreover, clinical and behavioral effects of genetic-risk disclosure have generally been reported to be minor or have tended to yield mixed results [36]. Given the absence of clear evidence for clinical utility or health benefits, DTC-GT proponents have put forward a broader case for societal benefits [37]. Alternatively, scientists involved in genetic research have stressed that the usefulness of genetic data lies in its crowdsourcing potential [38] and in having all data in one network [39]. Furthermore, the researchers behind the Personal Genome Project claim to have demonstrated that it is feasible for a research study to publicly share combined genomic and health data. They also found that when engaged in a participatory manner, contributors were highly motivated and volunteered their time and efforts to help create that data [40]. Other studies have also shown that users are indeed interested in DTC-GT in order to contribute to scientific research [41]. Our study supports this claim.

Given the uncertainties regarding the direct clinical benefits of DTC genetic testing, it could be hypothesized that individuals who publicly share their DTC-GT data may be seeking to extract other types of utility. This hypothesis is supported by our results, as a large majority of our respondents indicated that contributing to the advancement of medical research, wanting to help improve the predictability of genetic testing and considering it fun to explore genotype and phenotype data were relevant or very relevant motivations to share their data on openSNP.

Above all, however, our results suggest that health, even though prominent, did not seem to be the respondents’ primary or only motivation to be tested and share their data. In fact, we are dealing with a generally healthy population of respondents. And while several areas have been specifically articulated by the respondents (ancestry, scientific curiosity, health, contributing to research, nutrition) ancestry was most commonly mentioned in answers to questions on why users got tested in the first place and decided to share their data on openSNP. These results are consistent with the findings of a recent study on the motivations of DTC-GT user’s, which showed that the vast majority of users reported good to excellent health and that nonmedical information was of equal or greater interest to consumers [42].

Ancestry-related motivations deserve a closer look. Whereas it is generally assumed that purchasing genetic ancestry tests has a mainly recreational utility, consumers may also have other valuable personal motivations, as for example finding relatives or documenting one’s family history. Such uses, however, do not come without potential risks. Discovering bonds of kinship can be destabilizing, for both the person that purchased the test and those who get to be identified as relatives by someone they have never met before. In particular, discovering siblings through mitochondrial DNA or Y chromosome SNPs carries the potential to alter people’s perception of their personal identity and can, in consequence, turn out to be a source of deep psychological distress for individuals and their families alike.

Moreover, consumers also use genetic ancestry testing to learn about their racial and ethnic background–as in the case of African-Americans who seek for the geographical roots that slavery has obscured [43]. Yet when such uses stem from the need to negotiate one’s membership into a given social group, they may also lead to very tangible consequences, for instance with regard to college/university enrolment or employment. Establishing ethnicity through genotyping of a few allelic loci, however, is considerably less accurate than many consumers believe [44] and it is riddled with questionable assumptions about race and DNA. In particular, connecting racial and ethnic classifications to DNA obscures the historical, political and geographic determinants of such classifications. Finally, DTC genetic ancestry testing also lends itself to questionable political uses, as in the case of the Hungarian MP who resorted to a DTC-GT company to prove he does not have any “Roma or Jewish genes” [45]. Publicly sharing genetic and genomic data may thus amplify both the utilities and the risks of genetic ancestry testing.

### Data sharing and privacy risks

Over the past decade, IT companies have become the enablers and custodians of crucial technologies. They are increasingly operating as gatekeepers, and as a result occupy critical data junctions. DTC-GT companies are no exception. Indeed, genetic tests lead to crucial data and with that to new forms of power [46]. Therefore, the proliferation of DTC testing raises pressing questions about whether commercial firms are gaining access to health data without the necessary accountability. This is consistent with the circumstance that whereas the testing companies’ front-end activity consists in selling individual genetic tests online, their back-end business model involves amassing large privately owned genetic databases that they can exploit commercially [47]. As a result the privatization of genetic data has elicited novel anxieties and continues to raise many concerns regarding privacy, liability and consent [48,49]. As we report, however, whereas most of our respondents were well aware of the risks of their involvement in open genetic data sharing and consider the possibility of direct, personal repercussions troubling, they estimated the risk to be negligible. Our findings undermine a frequently entertained argument about the waning value that people allegedly attach to privacy in the era of connectivity and social media [50–52]. Quite to the contrary, our study shows that individuals are still concerned about privacy protection, even if they decide to share their genetic and genomic data publicly. It appears as though the respondents’ decision to openly share genetic data involves a certain compromise: to move forward in research, which is indeed a commonly-mentioned motivation for openSNP users, is to take a risk in the privacy and data-protection realm. Nonetheless, those who decide to take the risk, do so under the assumption that the risk is reasonably acceptable [53].

Certainly, understandings of risks can differ, particularly when–as in the case of genomics–there is no clear consensus about the likelihood of those risks. While some argue that there is very little holding testing companies to account, or that individual customers’ healthcare insurance premiums could go up, our survey has shown that health-related concerns are only of secondary interest to most customers who openly share their data. We thus expect that privacy concerns will not undermine the DTC testing industry, despite the regulatory constraints that emerged over the last few years to restrict the provision of health-related information to consumers.

### Data sharing and the right to participation

Until recently, genetic tests could only be obtained through healthcare providers. The latter would commission the appropriate analysis from a laboratory, collect and dispatch the samples and interpret the outcomes without involving the patient. Traditional research institutions, in turn, used to be the only players at the forefront of genetic research. Yet over the past decade, interest in genetic testing has spread far and wide beyond the conventional research realms, and often with growing intensity. Under a broadly conceived right to participation, companies have succeeded in accumulating health data from huge numbers of consumers, through DTC genetic tests sold online as well as through health and wellness apps and self–tracking devices [54]. In this respect, individuals, whose lives are ever more moulded by a wide range of mobile health applications or connected devices, are increasingly confronted by what has been called a perfect storm of information [55] to which they constantly contribute. Meanwhile, individuals keep gaining access to the technological means that allow them to take a more proactive role not only in their own health care but also as participants in the research process itself. Furthermore, the DTC-GT process commonly bypasses the involvement of healthcare professionals both upstream and downstream, as confirmed by data showing that the majority of subjects who had purchased a DTC-GT did not share their results with a health-care professional [56]. Nevertheless, some expect the proportion of users who share their results with a health-care professional to rise, once the opportunity presents itself or results become more pertinent to patients’ medical needs [57].

It has been argued that such recent developments have ushered in an era of increased scientific involvement for ordinary citizens, therefore opening new forms of engagement with science beyond the rather narrow possibilities afforded by participation in clinical research. In that sense, some citizens are operating outside of, or even antagonistically to, the traditional research environment–a phenomenon that has led some patients’ constituencies to conceptualize data sharing as a form of “disobedience” [58]. Arguably, platforms like openSNP stretch the meaning of participation a step further, as they enable users to mobilize their own data for research purposes. This feature, other than reshaping the notion of participation as an individual right, may also prelude to societal obligations to even promote such a right of individuals to use their personal and health data to conduct biomedical research outside the traditional scientific setting. While science used to be characterized by exclusivity, with traditional gatekeepers occupying all the critical junctions, the realm of genetic testing appears to undermine the scientific community’s once unchallenged authority. The convergence between, on the one hand, science and regulation and, on the other hand, a large public who traditionally held limited biomedical knowledge and power, begs further exploration.

## Conclusion

Throughout this paper we have shown that DTC-GT consumers who openly share their genetic data–far from focusing exclusively on the health-related aspects of genetic and genomic testing–are animated by diverse purposes and concerns. The present study thus contributes to a broader understanding of the issues pertaining to genetic and genomic data sharing and supports the idea that genomic utility should be understood across a variety of domains and perspectives. Instead of focusing exclusively on health-related aspects of genetic testing and data sharing, our study thus highlights that it is crucial to consider benefits, risks and developments that stretch beyond the health spectrum. Our findings support the call for a broader and multi-faceted conceptualization of genomic utility, one that pays due attention to the value users attach to being tested and to sharing data also in terms of contributing to the common good of research or seeking connection to other people and places [59]. As a consequence, such non-medical utilities, together with non-medical dis-utilities of the sort we mentioned above, should be an integral part of the ethical and regulatory debates surrounding DTC genetics and the practice of data sharing [60].

## Supporting information

S1 Text openSNP full questionnaire. (PDF)

S1 Table openSNP dataset. (XLS)

## References

[1] Lewin JH, Vis DJ, Voest EE, Liao R, Nederlof PM, Conley BA, et al Determining barriers to effective data sharing in cancer genomic sequencing initiatives: a global alliance for genomics and health (GA4GH) survey. Journal of Clinical Oncology. 2016;34(No 15_suppl):11502.

[2] Auffray C, Balling R, Barroso I, Bencze L, Benson M, Bergeron J, et al Making sense of big data in health research: towards an EU action plan. Genome Medicine. 2016;8(1):71 doi: 10.1186/s13073-016-0323-y 27338147 10.1186/s13073-016-0323-y PMC4919856

[3] The Global Alliance for Genomics and Health. A federated ecosystem for sharing genomic, clinical data. Science. 2016,352(6291):1278–80. doi: 10.1126/science.aaf6162 27284183 10.1126/science.aaf6162

[4] Rothstein MA, Knoppers BM, Harrell HL. Comparative approaches to biobanks and privacy. Journal of Law, Medicine & Ethics. 2016;44(1):161–72. 10.1177/1073110516644207 27256132

[5] Lapin V, Mighion L, da Silva C, Cuperus Y, Bean LJ, Hegde MR. Regulating whole exome sequencing as a diagnostic test. Human Genetics. 2016;135(6):655–73. doi: 10.1007/s00439-016-1677-3 27167135 10.1007/s00439-016-1677-3

[6] Shendure J, Mitra R, Varma C, Church G. Advanced sequencing technologies: methods and goals. Nature Reviews Genetics. 2004;5(5):335–44. doi: 10.1038/nrg1325 15143316 10.1038/nrg1325

[7] PGP Global Network [Internet]. Boston: PersonalGenomes.org; c2005-2016 [updated 2016 Nov 10; cited 2016 Dec 23]. AMA Office of Group Practice Liaison; [about 2 screens]. Personal Genome Project; [about 2 screens]: Available from: http://www.personalgenomes.org/organization/network

[8] Christofides E, O’Doherty K. Company disclosure and consumer perceptions of the privacy implications of direct-to-consumer genetic testing. New Genetics and Society 2016;35(2):101–123.

[9] dnatestingchoice.com [Internet]. London: Testing Choice Ltd.; c2013-16 [updated 2016 Nov 10; cited 2016 Dec 23]. Available from: https://dnatestingchoice.com

[10] dna.land [Internet]. New York: DNA.Land; c2015-16 [updated 2016 Nov 10; cited 2016 Dec 23]. Available from: https://dna.land

[11] Greshake B, Bayer P, Rausch H, Reda J. openSNP–a crowdsourced web resource for personal genomics. PLoS ONE. 2014;9(3):e89204 doi: 10.1371/journal.pone.0089204 24647222 10.1371/journal.pone.0089204 PMC3960092

[12] opensnp.org [Internet]. Offenbach am Main (Germany): OpenSNP Org; c2017 [cited 2017 March 7]. Available from: https://opensnp.org/signup.

[13] openSNP [dataset]. March 7, 2017 [cited 2017 March 7]. Available from: openSNP.org

[14] Humbert M, Huguenin K, Hugonot J, Ayday E, Hubaux JP. De-anonymizing genomic databases using phenotypic traits. Proceedings on Privacy Enhancing Technologies. 2015;2: 99–114.

[15] Corpas M. Further bias in personal genomics? Front Line Genomics [Internet]. 2016 Oct cited 2017 Feb 8]. Available from: http://www.frontlinegenomics.com/opinion/8051/further-bias-personal-genomics/

[16] Hartung AM. Investigation of methods for machine learning associations between genetic variations and phenotype [dissertation]. Rochester (NY): Rochester Institute of Technology; 2016. Available from: http://scholarworks.rit.edu/theses/9185

[17] Zaaijer S, Gordon A, Piccone R, Speyer D, Erlich Y. Democratizing DNA fingerprinting. bioRxiv preprint. 2016 Jun 30.

[18] McGuire AL, Hamilton JA, Lunstroth R, McCullough LB, Goldman A. DNA data sharing: research participants' perspectives. Genetics in Medicine. 2008;10:46–53. doi: 10.1097/GIM.0b013e31815f1e00 18197056 10.1097/GIM.0b013e31815f1e00 PMC2767246

[19] Brown Trinidad S, Fullerton SM, Bares JM, Jarvik GP, Larson EB, Burke W. Genomic research and wide data sharing: views of prospective participants. Genetics in Medicine. 2010;12:486–95. doi: 10.1097/GIM.0b013e3181e38f9e 20535021 10.1097/GIM.0b013e3181e38f9e PMC3045967

[20] Lemke AA, Wolf WA, Hebert-Beirne J, Smith ME. Public and biobank participant attitudes toward genetic research participation and data sharing. Public Health Genomics. 2010;13:368–77. doi: 10.1159/000276767 20805700 10.1159/000276767 PMC2951726

[21] Oliver JM, Slashinski MJ, Wang T, Kelly PA, Hilsenbeck SG, McGuire AL. Balancing the risks and benefits of genomic data sharing: genome research participants’ perspectives. Public Health Genomics. 2012;15:106–14. doi: 10.1159/000334718 22213783 10.1159/000334718 PMC3318928

[22] Wallis JC, Rolando E, Borgman CL. If we share data, will anyone use them? Data sharing and reuse in the long tail of science and technology. PLoS ONE. 2013;8(7):e67332 doi: 10.1371/journal.pone.0067332 23935830 10.1371/journal.pone.0067332 PMC3720779

[23] Ball M, Bobe J, Chou M, Clegg T, Estep PW, Lunshof JE, et al Harvard Personal Genome Project: lessons from participatory public research. Genome Medicine. 2014;6(2):10 doi: 10.1186/gm527 24713084 10.1186/gm527 PMC3978420

[24] Critchley C, Nicol D, Otlowski M. The Impact of commercialisation and genetic data sharing arrangements on public trust and the intention to participate in biobank research. Public Health Genomics. 2015;18:160–72. doi: 10.1159/000375441 25790760 10.1159/000375441

[25] Cheung C, Bietz MJ, Patrick K, Bloss CS. Privacy attitudes among early adopters of emerging health technologies. PLoS ONE. 2016;11(11):e0166389 doi: 10.1371/journal.pone.0166389 27832194 10.1371/journal.pone.0166389 PMC5104519

[26] Tse-Hua S, Fan X. Comparing response rates from web and mail surveys: a meta-analysis. Field Methods. 2008;20(3):249–71.

[27] openSNP [dataset]. February 6, 2017 [cited 2017 Feb 6]. Available from: openSNP.org

[28] Carere DA, VanderWeele TJ, Vassy JL, van der Wouden CH, Roberts JS, Kraft P, et al Prescription medication changes following direct-to-consumer personal genomic testing: findings from the impact of Personal Genomics (PGen) Study. Genetics in Medicine. 2016. Epub 2016 Sep 22. 10.1038/gim.2016.141 PMC5362351 27657683

[29] Roberts JS, Gornick MC, Carere DA, Uhlmann WR, Ruffin MT, Green RC. Direct-to-consumer genetic testing: user motivations, decision making, and perceived utility of results. Public Health Genomics. 2017. Epub 2017 Jan 10. 10.1159/000455006 PMC12834086 28068660

[30] Annas G, Elias S. 23andMe and the FDA. New England Journal of Medicine. 2014; 370(11):985–88. doi: 10.1056/NEJMp1316367 24520936 10.1056/NEJMp1316367

[31] Biotechnology Nature. FDA approves 23andMe gene carrier test. Nature Biotechnology. 2015;33(5):435. 10.1038/nbt0515-435a 25965732

[32] Vayena E. Direct-to-consumer genomics on the scales of autonomy. Journal of Medical Ethics. 2014;41 (4):310–14. doi: 10.1136/medethics-2014-102026 24797610 10.1136/medethics-2014-102026 PMC4392219

[33] Roberts J, Ostergren J. Direct-to-consumer genetic testing and personal genomics services: A review of recent empirical studies. Current Genetic Medicine Reports. 2013; 1(3):182–200. 24058877 10.1007/s40142-013-0018-2 PMC3777821

[34] Gray SW, Gollust SE, Carere DA, Chen CA, Cronin A, Kalia SS, et al Personal genomic testing for cancer risk: results from the impact of personal genomics study. J Clin Oncol. 2016. Epub 2016 Dec 12. 10.1200/JCO.2016.67.1503 PMC5455805 27937091

[35] Bloss CS, Schork NJ, Topol EJ. Effect of direct-to-consumer genomewide profiling to assess disease risk. New England Journal of Medicine. 2011;364:524–34. doi: 10.1056/NEJMoa1011893 21226570 10.1056/NEJMoa1011893 PMC3786730

[36] Heshka JT, Palleschi C, Howley H, Wilson B, Wells PS. A systematic review of perceived risks, psychological and behavioral impacts of genetic testing. Genetics in Medicine. 2008;10:19–32. doi: 10.1097/GIM.0b013e31815f524f 18197053 10.1097/GIM.0b013e31815f524f

[37] Nelson B. Greater goods?: direct-to-consumer testing companies are making a broader case for societal benefits, but not everyone is sold. Cancer Cytopathology. 2016;124(3):159–60. doi: 10.1002/cncy.21706 26972119 10.1002/cncy.21706

[38] Corpas M, Valdivia-Granda W, Torres N, Greshake B, Coletta A, Knaus A, et al Crowdsourced direct-to-consumer genomic analysis of a family quartet. BMC Genomics. 2015;16(1):910. 26547235 10.1186/s12864-015-1973-7 PMC4636840

[39] Greshake B, Bayer P, Rausch H, Reda J. openSNP–a crowdsourced web resource for personal genomics. PLoS ONE. 2014;9(3):e89204 doi: 10.1371/journal.pone.0089204 24647222 10.1371/journal.pone.0089204 PMC3960092

[40] Ball M, Bobe J, Chou M, Clegg T, Estep PW, Lunshof JE, et al Harvard Personal Genome Project: lessons from participatory public research. Genome Medicine. 2014;6(2):10 doi: 10.1186/gm527 24713084 10.1186/gm527 PMC3978420

[41] Vayena E, Ineichen C, Stoupka E, Hafen E. Playing a part in research? University students' attitudes to direct-to-consumer genomics. Public Health Genomics. 2014;17(3):158–68. doi: 10.1159/000360257 24777115 10.1159/000360257

[42] Roberts JS, Gornick MC, Carere DA, Uhlmann WR, Ruffin MT, Green RC. Direct-to-consumer genetic testing: user motivations, decision making, and perceived utility of results. Public Health Genomics. 2017. Epub 2017 Jan 10. 10.1159/000455006 PMC12834086 28068660

[43] Rotimi C. Genetic ancestry tracing and the African identity: a double-edged sword?. Developing World Bioethics. 2003;3(2):151–58. 14768647 10.1046/j.1471-8731.2003.00071.x

[44] Royal C, Novembre J, Fullerton S, Goldstein DB, Long JC, Bamshad MJ, et al Inferring genetic ancestry: opportunities, challenges, and implications. The American Journal of Human Genetics. 2010;86(5):661–73. doi: 10.1016/j.ajhg.2010.03.011 20466090 10.1016/j.ajhg.2010.03.011 PMC2869013

[45] Abbott A. Genome test slammed for assessing ‘racial purity’. Nature. 2012;486:167 doi: 10.1038/486167a 22699587 10.1038/486167a

[46] Wilbanks JT, Topol EJ. Privacy: Stop the privatization of health data. Nature [Internet]. 2016 7 [cited 2017 Feb 10]. Available from: http://www.nature.com/nature/journal/v537/n7619_supp/full/537S70a.html 10.1038/535345a 27443724

[47] Spector-Bagdady K. “The Google of Healthcare”: enabling the privatization of genetic bio/databanking. Annals of Epidemiology. 2016;26(7):515–19. doi: 10.1016/j.annepidem.2016.05.007 27449572 10.1016/j.annepidem.2016.05.007 PMC6988384

[48] Laestadius L, Rich J, Auer P. All your data (effectively) belong to us: data practices among direct––to-consumer genetic testing firms. Genetics in Medicine. 2016. Epub 2016 Sep 22. 10.1038/gim.2016.136 27657678

[49] Slavkovic A, Yu F. O privacy, where art thou? Genomics and privacy. CHANCE. 2015;28(2):37–9.

[50] Sprenger P. Sun on privacy: 'get over it'. WIRED. [Internet]. 1999 Jan [cited 2017 Feb 10]. Available from: http://archive.wired.com/politics/law/news/1999/01/17538

[51] Tubaro P, Casilli AA, Yasaman S. Against the hypothesis of the end of privacy: an agent-based modelling approach to social media. London: Springer; 2014.

[52] Savage N. Privacy: the myth of anonymity. Nature [Internet]. 2016 9 [cited 2017 Feb 10];537:[about 2 p.]. Available from: http://www.nature.com/nature/journal/v537/n7619_supp/full/537S70a.html 10.1038/537S70a 27602747

[53] Evans J and Green R. Direct to consumer genetic testing: avoiding a culture war. Genetics in Medicine. 2009;11(8):568–69. doi: 10.1097/GIM.0b013e3181afbaed 19606051 10.1097/GIM.0b013e3181afbaed PMC2920210

[54] Vayena E, Mastroianni A, Kahn J. Ethical issues in health research with novel online sources. American Journal of Public Health. 2012;102(12):2225–30. doi: 10.2105/AJPH.2012.300813 23078484 10.2105/AJPH.2012.300813 PMC3519346

[55] Fernández-Luque L, Bau T. Health and social media: perfect storm of information. Healthcare Informatics Research. 2015;21(2):67 doi: 10.4258/hir.2015.21.2.67 25995958 10.4258/hir.2015.21.2.67 PMC4434065

[56] McGrath S, Coleman J, Najjar L, Fruhling A, Bastola DR. Comprehension and data-sharing behavior of direct-to-consumer genetic test customers. Public Health Genomics. 2016;19(2):116–24. doi: 10.1159/000444477 26950077 10.1159/000444477

[57] van der Wouden CH, Carere DA, Maitlandvan der Zee AH, Ruffin MT, Roberts JC, Green RC. Consumer perceptions of interactions with primary care providers after direct-to-consumer personal genomic testing. Ann Intern Med, 2016;164:513–22. doi: 10.7326/M15-0995 26928821 10.7326/M15-0995

[58] Wicks P, Vaughan T, Heywood J. Subjects no more: what happens when trial participants realize they hold the power? BMJ. 2014;348:g368 doi: 10.1136/bmj.g368 24472779 10.1136/bmj.g368 PMC3905107

[59] Chung MW, Ng JC. Personal utility is inherent to direct-to-consumer genomic testing. Journal of Medical Ethics. 2016;42(10):649–52. doi: 10.1136/medethics-2015-103057 27250638 10.1136/medethics-2015-103057

[60] Vayena E, Gourna E, Streuli J, Hafen E, Prainsack B. Experiences of early users of direct-to-consumer genomics in Switzerland: an exploratory study. Public Health Genomics. 2012;15(6):352–62. doi: 10.1159/000343792 23154382 10.1159/000343792

