# Supplementary material for: Open sharing of genomic data: Who does it and why?
Source: PLoS One. 2017 May 9;12(5):e0177158. doi: 10.1371/journal.pone.0177158 (PMC5423632; doi:10.1371/journal.pone.0177158)
Supplement: S1 Text — (PDF) [file pone.0177158.s001.pdf]

Dear openSNP user,

WELCOME TO THE SURVEY ABOUT GENETIC DATA SHARING

What is your gender?

- ○ female
- ○ male
- ○ other

What is your year of birth?

What is the highest degree or level of education you have completed?

- ○ less than high school
- ○ high school graduate
- ○ bachelor's degree
- ○ master's degree
- ○ doctorate (Ph.D.)

Do you have children?

- ○ no
- ○ yes

Are you engaged with biomedical research? (multiple answers possible)

- ☐ no
- ☐ yes, as a professional
- ☐ yes, as a research participant

Yes, in another function. Please specify:

In which country do you live?

Have you ever tested your DNA/genotype profile

- ◯ no
- ◯ yes

Which service(s) did you use to test your DNA/genotype profile? (multiple answers possible)

- [ ] 23andMe
- [ ] bio.logis
- [ ] deCODEme
- [ ] GenePlanet
- [ ] GeneleX
- [ ] Pathway Genomics
- [ ] Navigenics
- [ ] dna.ancestry

Other services, please specify:

[ ]

What were your interests in being tested? Please, rank the following options.

Rank	Option	
[ ]	Ancestry	[ ] was not relevant
[ ]	Health	[ ] was not relevant
[ ]	Contribution to research	[ ] was not relevant

Please specify any other interests you may had in being tested:

[ ]

Has your DNA analysis helped you to improve your health or life style?

- ( ) not at all
- ( ) to a small extent
- ( ) to a moderate extent
- ( ) to a large extent

Do you have your genotype raw-data uploaded on openSNP?

- ◯ no
- ◯ yes

How long ago did you upload your genotype raw-data?

- ◯ more than two years ago
- ◯ a few months ago but less than two years
- ◯ a few weeks ago

How often do you visit openSNP?

- ◯ I visited openSNP only for uploading data
- ◯ I visited openSNP a few times since data upload
- ◯ I visit openSNP occasionally
- ◯ I visit openSNP regularly

In addition to your genotype raw-data, did you upload other data on openSNP?(multiple answers possible)

- ☐ no, I am not interested in uploading other data
- ☐ no, not yet but probably in the future
- ☐ yes, phenotypes
- ☐ yes, ZIP code
- ☐ yes, profile picture

Yes, other data, please specify:

Do you have further comments about what data you have chosen to upload or not to upload?

Have you yourself already created one or more new phenotype(s)?

- ◯ no, I am not interested in creating phenotypes
- ◯ no, not yet but probably in the future
- ◯ yes

Have you ever contacted other users by use of the messaging function on openSNP?

- ◯ no, I am not interested in contacting other users
- ◯ no, not yet but probably in the future
- ◯ yes, I have contacted only one person
- ◯ yes, I have contacted several people

Have you ever met someone in person with whom you corresponded via*openSNP*?

- ◯ no, I am not interested in meeting other users
- ◯ no, but I would definitely like to
- ◯ no, not yet but probably in the future
- ◯ yes

Have you ever downloaded data from openSNP to conduct own research or statistical analysis?

- ◯ no, I am not interested in downloading data to conduct analyses
- ◯ no, not yet but probably in the future
- ◯ yes

How carefully did you read the terms and conditions for sharing data while registering on openSNP?

- ◯ I did not read them at all
- ◯ I only read them over quickly
- ◯ I read them carefully

Have you ever considered deleting your profile or data on openSNP?

- ◯ no
- ◯ yes

Do you share your genotype raw-data in other networks? (multiple answers possible)

- [ ] no
- [ ] Personal Genome Project
- [ ] Genomera
- [ ] OpenHumans
- [ ] DNA.land
- [ ] Your own personal website

In other networks, please specify:

Do you have a publicly accessible profile on social networks? (multiple answers possible)

- [ ] no
- [ ] yes, on Facebook
- [ ] yes, on Twitter
- [ ] yes, on Google+
- [ ] yes, on PatientsLikeMe

Yes, on other social networks, please specify:

At the time when you decided to upload data on*openSNP*:

Did you speak with others before making the decision to join openSNP? (multiple answers possible)

- ☐ no
- ☐ yes, with my partner
- ☐ yes, with family members
- ☐ yes, with friends
- ☐ yes, with my physician or a geneticist

Yes, with others, please specify:

How difficult was the decision to make?

- ○ very easy
- ○ rather easy
- ○ rather difficult
- ○ very difficult

Why did you decide to share data on openSNP?

Indicate how relevant the following reasons were for your decision

	not at all relevant	somewhat relevant	relevant	very relevant
It seemed fun to explore genotype and phenotype data	○	○	○	○
I wanted to help improve the predictability of genetic testing	○	○	○	○
I wanted to contribute to the advancement of medical research	○	○	○	○
I wanted to feel as a helpful person	○	○	○	○
I thought other users or the scientific community would be appreciative if I shared my data with them	○	○	○	○
I wanted to learn more about myself	○	○	○	○
I wanted to make more use of my genotype data by combining them with my phenotype data	○	○	○	○
I wanted to get in contact with people who are similar to me	○	○	○	○
I wanted to find people who understand me	○	○	○	○
I wanted to find my “soulmate“	○	○	○	○
Sharing my data seemed like a good way to handle my anxieties about my genetic test results and health risks	○	○	○	○
I sought reassurance that other people might share some specific genotype or phenotype data with me	○	○	○	○
I wanted to have influence on research	○	○	○	○
I wanted to gain insights for my own research	○	○	○	○

Did you upload your data for other reasons? Or do you have any other comments about your decision? Please specify:

Imagine the following scenario:

Your genomic data on openSNP are not only used for academic research purposes.

Do you think this is likely to happen?

- ◯ very unlikely
- ◯ rather unlikely
- ◯ rather likely
- ◯ very likely

How concerned would you be, if this was to happen?

- ◯ not at all concerned
- ◯ rather not concerned
- ◯ rather concerned
- ◯ very concerned

Imagine the following scenario:

Your genomic data on openSNP are used by commerical purposes for marketing.

Do you think this is likely to happen?

- ◯ very unlikely
- ◯ rather unlikely
- ◯ rather likely
- ◯ very likely

How concerned would you be, if this was to happen?

- ◯ not at all concerned
- ◯ rather not concerned
- ◯ rather concerned
- ◯ very concerned

Imagine the following scenario:

Once put on openSNP, your genetic data will continue to exist somewhere and they can never be fully deleted.

Do you think this is likely to happen?

- ◯ very unlikely
- ◯ rather unlikely
- ◯ rather likely
- ◯ very likely

How concerned would you be, if this was to happen?

- ◯ not at all concerned
- ◯ rather not concerned
- ◯ rather concerned
- ◯ very concerned

Imagine the following scenario:

Research that you would not approve of is done with your data on openSNP.

Do you think this is likely to happen?

- ◯ very unlikely
- ◯ rather unlikely
- ◯ rather likely
- ◯ very likely

How concerned would you be, if this was to happen?

- ◯ not at all concerned
- ◯ rather not concerned
- ◯ rather concerned
- ◯ very concerned

Can you think of a particular type of research you would object to? Please specify:

Imagine the following scenario:

Some of your relatives are identified using your data on openSNP.

Do you think this is likely to happen?

- ○ very unlikely
- ○ rather unlikely
- ○ rather likely
- ○ very likely

How concerned would you be, if this was to happen?

- ○ not at all concerned
- ○ rather not concerned
- ○ rather concerned
- ○ very concerned

Imagine the following scenario:

Your genotype data on openSNP are used to your disadvantage by an employer.

Do you think this is likely to happen?

- very unlikely
- rather unlikely
- rather likely
- very likely

How concerned would you be, if this was to happen?

- not at all concerned
- rather not concerned
- rather concerned
- very concerned

Imagine the following scenario:

Your genotype data on openSNP are used to your disadvantage by an insurance company.

Do you think this is likely to happen?

- ◯ very unlikely
- ◯ rather unlikely
- ◯ rather likely
- ◯ very likely

How concerned would you be, if this was to happen?

- ◯ not at all concerned
- ◯ rather not concerned
- ◯ rather concerned
- ◯ very concerned

Imagine the following scenario:

You receive negative comments by other users on openSNP.

Has this happened to you?

- ◯ yes, this has happened
- ◯ no, this has not happened

Imagine the following scenario:

You receive negative comments by other users on openSNP.

How concerned are you about this?

- ◯ not at all concerned
- ◯ rather not concerned
- ◯ rather concerned
- ◯ very concerned

Imagine the following scenario:

You receive negative comments by other users on openSNP.

Do you think this is likely to happen?

- ○ very unlikely
- ○ rather unlikely
- ○ rather likely
- ○ very likely

How concerned would you be, if this was to happen?

- ○ not at all concerned
- ○ rather not concerned
- ○ rather concerned
- ○ very concerned

Imagine the following scenario:

After you have shared your data on openSNP, medical research reveals a previously unknown association between gene variants and a disease that you would rather not know about.

Has this happened with your gene variants?

- ◯ yes, this has happened
- ◯ no, this has certainly not happened
- ◯ not that I am aware

Imagine the following scenario:

After you have shared your data on openSNP, medical research reveals a previously unknown association between gene variants and a disease that you would rather not know about.

How concerned are you about this?

- ◯ not at all concerned
- ◯ rather not concerned
- ◯ rather concerned
- ◯ very concerned

Imagine the following scenario:

After you have shared your data on openSNP, medical research reveals a previously unknown association between gene variants and a disease that you would rather not know about.

Do you think this is likely to happen?

- very unlikely
- rather unlikely
- rather likely
- very likely

How concerned would you be, if this was to happen?

- not at all concerned
- rather not concerned
- rather concerned
- very concerned

Imagine the following scenario:

You are irritated or made feel uncomfortable by other users on openSNP or researchers that are interested in your profile.

Has this happened to you?

- ◯ yes, this has happened
- ◯ no, this has not happened

Imagine the following scenario:

You are irritated or made feel uncomfortable by other users on openSNP or researchers that are interested in your profile.

How concerned are you about this?

- not at all concerned
- rather not concerned
- rather concerned
- very concerned

Imagine the following scenario:

You are irritated or made feel uncomfortable by other users on openSNP or researchers that are interested in your profile.

Do you think this is likely to happen?

- ◯ very unlikely
- ◯ rather unlikely
- ◯ rather likely
- ◯ very likely

How concerned would you be, if this was to happen?

- ◯ not at all concerned
- ◯ rather not concerned
- ◯ rather concerned
- ◯ very concerned

To what extent has your sharing of data on openSNP resulted in the following experiences

	not at all true	rather not true	rather true	very true
It is/was fun to explore genotype and phenotype data	◯	◯	◯	◯
I feel that I can help improve the predictability of genetic testing	◯	◯	◯	◯
I feel that I can contribute to the advance of medical research	◯	◯	◯	◯
I feel that I am a helpful person	◯	◯	◯	◯
I feel that other users or the scientific community do appreciate that I share data with them	◯	◯	◯	◯
I am learning about myself	◯	◯	◯	◯
My genotype data is more useful for me when combined with my phenotype variations	◯	◯	◯	◯
I have got in contact with people who are similar to me	◯	◯	◯	◯
I have found people who understand me	◯	◯	◯	◯
I found my “soulmate“	◯	◯	◯	◯
Sharing my data helped me handle my worries about my genetic tests and health risks	◯	◯	◯	◯
It comforts/comforted me to see that other people share some specific genotypical or phenotypical data with me	◯	◯	◯	◯
I feel that I have influenced research	◯	◯	◯	◯
I have gained insights for my own research	◯	◯	◯	◯

Are there any other positive or negative experiences since sharing your data on openSNP? Please specify:

In general, how satisfied are you with your data sharing on openSNP?

- very unsatisfied
- rather unsatisfied
- rather satisfied
- very satisfied

Do you feel that you are more or less in control of your genetic data since you uploaded them on openSNP?

- much less in control
- less in control
- the same as before data sharing
- more in control
- much more in control

Would you recommend others to share data on openSNP?

- no
- yes

Why wouldn't you recommend it to others?

☐ because of the negative experiences that I indicated earlier on

Please feel free to describe your negative experiences in detail or to provide other reasons:

Why would you recommend it to others?

☐ because of the positive experiences that I indicated earlier on

Please feel free to describe your positive experiences in detail or to provide other reasons:

Genetic data sharing can raise concerns about privacy and data protection. How would you rate the following statements?

	strongly disagree	disagree	agree	strongly agree	no opinion
I am more than my genes. Genetic data are just biological parameters. There are definitely other personal data that I care more about.	◯	◯	◯	◯	◯
Privacy/data protection is anyway an illusion in the digital age—let’s get over it.	◯	◯	◯	◯	◯
Genetic data are like a fingerprint, they clearly identify people—their protection is very important	◯	◯	◯	◯	◯
In the big data era we live in, protection of all kinds of personal data should be taken very seriously	◯	◯	◯	◯	◯
Privacy is too abstract a notion—it hardly means something to me	◯	◯	◯	◯	◯
The idea of the “transparent citizen“ frightens me	◯	◯	◯	◯	◯
Taking privacy risks may be justified by the benefits of medical research	◯	◯	◯	◯	◯
Privacy concerns reflect concervatism. People should be more open to new technologies and innovation	◯	◯	◯	◯	◯
I have done nothing wrong, so I have noting to hide	◯	◯	◯	◯	◯

Have you ever considered to upload your genotype raw-data on openSNP?

- ◯ no
- ◯ yes

Do you share your genotype raw-data in other networks? (multiple answers possible)

- ☐ no
- ☐ Personal Genome Project
- ☐ Genomera
- ☐ OpenHumans
- ☐ DNA.land
- ☐ Your own personal website

In other networks, please specify:

How long ago did you register with openSNP?

- ◯ more than two years ago
- ◯ a few months ago but less than two years
- ◯ a few weeks ago

How often do you visit openSNP?

- ◯ I visited *openSNP* only for registering
- ◯ I visited *openSNP* a few times since registration
- ◯ I visit *openSNP* occasionally
- ◯ I visit *openSNP* regularly

Have you ever downloaded data from openSNP to conduct own research or statistical analysis?

- ◯ no, I am not interested in downloading data to conduct analyses
- ◯ no, not yet but probably in the future
- ◯ yes

How carefully did you read the terms and conditions for sharing data while registering on openSNP?

- ◯ I did not read them at all
- ◯ I only read them over
- ◯ I read them carefully

Have you ever considered deleting your profile on openSNP?

- ◯ no
- ◯ yes

Do you have a publicly accessible profile on social networks? (multiple answers possible)

- [ ] no
- [ ] yes, on Facebook
- [ ] yes, on Twitter
- [ ] yes, on Google+
- [ ] yes, on PatientsLikeMe

Yes, on other social networks, please specify:

Do you value the following possible benefits of sharing genotype data on openSNP?

	not at all	a little bit	somewhat	very much
The fun of exploring genotype and phenotype data	◯	◯	◯	◯
Improving the predictability of genetic testing	◯	◯	◯	◯
Contributing to the advancement of medical research	◯	◯	◯	◯
The feeling of being a helpful person	◯	◯	◯	◯
Being appreciated by other users or the scientific community for sharing data with them	◯	◯	◯	◯
Learning more about myself	◯	◯	◯	◯
Making more use of my genotype data by combining them with my phenotype data	◯	◯	◯	◯
Getting in contact with people who are similar to me	◯	◯	◯	◯
Finding people who understand me	◯	◯	◯	◯
Finding my “soulmate“	◯	◯	◯	◯
Handling anxieties about genetic test results and health risks	◯	◯	◯	◯
Seeking reassurance that other people might share some specific genotype or phenotype data with me	◯	◯	◯	◯
Influencing research	◯	◯	◯	◯
Gaining insights for my own research	◯	◯	◯	◯

Imagine the following scenario:

Genomic data on openSNP are not only used for academic research purposes.

Do you think this is likely to happen?

- ◯ very unlikely
- ◯ rather unlikely
- ◯ rather likely
- ◯ very likely
- ◯ I have no opinion

How concerned would you be, if you shared data and this was to happen?

- ◯ not at all concerned
- ◯ rather not concerned
- ◯ rather concerned
- ◯ very concerned

Imagine the following scenario:

Genomic data on openSNP are used by commerical purposes for marketing.

Do you think this is likely to happen?

- ◯ very unlikely
- ◯ rather unlikely
- ◯ rather likely
- ◯ very likely
- ◯ I have no opinion

How concerned would you be, if you shared data and this was to happen?

- ◯ not at all concerned
- ◯ rather not concerned
- ◯ rather concerned
- ◯ very concerned

Imagine the following scenario:

Once put on openSNP, genetic data will continue to exist somewhere and they can never be fully deleted.

Do you think this is likely to happen?

- ◯ very unlikely
- ◯ rather unlikely
- ◯ rather likely
- ◯ very likely
- ◯ I have no opinion

How concerned would you be, if you shared data and this was to happen?

- ◯ not at all concerned
- ◯ rather not concerned
- ◯ rather concerned
- ◯ very concerned

Imagine the following scenario:

Research that people do not approve of is done with their data on openSNP.

Do you think this is likely to happen?

- ◯ very unlikely
- ◯ rather unlikely
- ◯ rather likely
- ◯ very likely
- ◯ I have no opinion

How concerned would you be, if you shared data and this was to happen?

- ◯ not at all concerned
- ◯ rather not concerned
- ◯ rather concerned
- ◯ very concerned

Can you think of a particular type of research you would object to? Please specify:

Imagine the following scenario:

Relatives of those who share data are identified using data on openSNP.

Do you think this is likely to happen?

- ◯ very unlikely
- ◯ rather unlikely
- ◯ rather likely
- ◯ very likely
- ◯ I have no opinion

How concerned would you be, if you shared data and this was to happen?

- ◯ not at all concerned
- ◯ rather not concerned
- ◯ rather concerned
- ◯ very concerned

Imagine the following scenario:

Genotype data on openSNP are used to the disadvantage of those who share data by employers.

Do you think this is likely to happen?

- ◯ very unlikely
- ◯ rather unlikely
- ◯ rather likely
- ◯ very likely
- ◯ I have no opinion

How concerned would you be, if you shared data and this was to happen?

- ◯ not at all concerned
- ◯ rather not concerned
- ◯ rather concerned
- ◯ very concerned

Imagine the following scenario:

Genotype data on openSNP are used against those who share data by insurance companies.

Do you think this is likely to happen?

- ◯ very unlikely
- ◯ rather unlikely
- ◯ rather likely
- ◯ very likely
- ◯ I have no opinion

How concerned would you be, if you shared data and this was to happen?

- ◯ not at all concerned
- ◯ rather not concerned
- ◯ rather concerned
- ◯ very concerned

Imagine the following scenario:

Those who share data receive negative comments by other users on openSNP.

Do you think this is likely to happen?

- ◯ very unlikely
- ◯ rather unlikely
- ◯ rather likely
- ◯ very likely
- ◯ I have no opinion

How concerned would you be, if you shared data and this was to happen?

- ◯ not at all concerned
- ◯ rather not concerned
- ◯ rather concerned
- ◯ very concerned

Imagine the following scenario:

After one has shared data on openSNP, medical research reveals a previously unknown association between gene variants and a disease that the sharing person would rather not know about.

Do you think this is likely to happen?

- ○ very unlikely
- ○ rather unlikely
- ○ rather likely
- ○ very likely
- ○ I have no opinion

How concerned would you be, if you shared data and this was to happen?

- ○ not at all concerned
- ○ rather not concerned
- ○ rather concerned
- ○ very concerned

Imagine the following scenario:

One is irritated or made feel uncomfortable by other users on openSNP or researchers that are interested in one's profile.

Do you think this is likely to happen?

- very unlikely
- rather unlikely
- rather likely
- very likely
- I have no opinion

How concerned would you be, if you shared data and this was to happen?

- not at all concerned
- rather not concerned
- rather concerned
- very concerned

Genetic data sharing can raise concerns about privacy and data protection. How would you rate the following statements?

	strongly disagree	disagree	agree	strongly agree	no opinion
I am more than my genes. Genetic data are just biological parameters. There are definitely other personal data that I care more about.	◯	◯	◯	◯	◯
Privacy/data protection is anyway an illusion in the digital age—let's get over it.	◯	◯	◯	◯	◯
Genetic data are like a fingerprint, they clearly identify people—their protection is very important	◯	◯	◯	◯	◯
In the big data era we live in, protection of all kinds of personal data should be taken very seriously	◯	◯	◯	◯	◯
Privacy is too abstract a notion—it hardly means something to me	◯	◯	◯	◯	◯
The idea of the "transparent citizen" frightens me	◯	◯	◯	◯	◯
Taking privacy risks may be justified by the benefits of medical research	◯	◯	◯	◯	◯
Privacy concerns reflect concervatism. People should be more open to new technologies and innovation	◯	◯	◯	◯	◯
I have done nothing wrong, so I have noting to hide	◯	◯	◯	◯	◯

Thank you for completing the survey.

In the next step of the project, we would like to conduct in-depth interviews on the subject to gain more insights.

If you are willing to share your experiences in an interview, please provide us your contact details in the text field below or email us: HEPLab@ebpi.uzh.ch

We very much appreciate your participation.

Your contact details:

Please, click on «done» to finish the survey.
